# Exclusion Criteria Used in Early Behavioral Intervention Studies for Young Children with Autism Spectrum Disorder

**DOI:** 10.3390/brainsci10020099

**Published:** 2020-02-13

**Authors:** Sahr Yazdani, Angela Capuano, Mohammad Ghaziuddin, Costanza Colombi

**Affiliations:** 1 Stritch School of Medicine, Loyola University, Maywood, IL 60153, USA; syazdani1@luc.edu; 2 Department of Psychiatry, University of Michigan, Ann Arbor, MI 48109, USA; acapuano@umich.edu (A.C.); mghaziud@umich.edu (M.G.)

**Keywords:** autism spectrum disorder, autism, literature review, comorbidity, early intervention, early intensive behavioral intervention, behavioral intervention

## Abstract

This literature review evaluated early behavioral intervention studies of Autism Spectrum disorder (ASD) based on their participant exclusion criteria. The studies included were found through searching PsycINFO and PubMed databases, and discussed behavioral interventions for children up to 5 years of age with ASD and utilized a group research design. Studies reviewed were categorized into three groups: Restrictive exclusion criteria, loosely defined exclusion criteria, and exclusion criteria not defined. Results indicated that studies that used restrictive exclusion criteria demonstrated greater differences in terms of outcomes between experimental and control groups in comparison to studies that used loosely defined exclusion criteria and/or did not define any exclusion criteria. We discussed implications for the generalizability of the studies’ outcomes in relationship to exclusion criteria.

## 1. Introduction

Autism Spectrum Disorder (ASD) is a neurodevelopmental disorder that involves impairments in social communication, as well as the presence of stereotyped patterns of behaviors and interests [1]. ASD is considered a leading cause of disability in children under 5 years of age [2]. Given that ASD affects approximately 1 in 59 children in the United States [3], it is considered a serious public health concern [4]. This higher prevalence may be partially due to better detection and assessment procedures and an expanded definition of ASD [3,5,6]. 

While in the past, children with ASD were typically diagnosed around the age of 4 years shortly before entering school, they are now being diagnosed as early as the age of 2 years [7,8] and identified as at-risk for ASD between 12 to 24 mon of age [9]. With the increase in the number of young children being diagnosed, developing early age-appropriate interventions that can support parents and children is an international clinical and research priority [10,11].

Currently, research evidence indicates that high-intensity, long-term behavioral interventions are the most efficacious in supporting development and diminishing ASD symptoms and associated disabilities [12,13,14,15,16,17]. In a seminal study on behavioral intervention for children with ASD, Lovaas [14] demonstrated that children aged 40 to 46 mon who participated in intensive, long-term applied behavior analysis therapy achieved remarkable improvement in their skills. Specifically, nearly half of the children enrolled in intensive applied behavior analysis (for a minimum of 40 h per week), for at least 2 years showed significant gains in their adaptive and intellectual functioning, with some children becoming nearly indistinguishable from their typically developing peers. At long-term follow-up, the children who made significant gains maintained those gains, with placement in mainstream classrooms. This study led to widespread interest in behavioral interventions as promising treatments for children with ASD, spurring the development of educational treatment programs [18]. 

Despite the promising results found in the Lovaas [14] study, there was variability in the functioning of the study participants, with 40% of the participants continuing to meet criteria for developmental delays and needing educational supports. Replication of the Lovaas [14] study provided partial support for the treatment gains achieved, but with some disappointing results as the gains made during the replication were not as robust as the original study [18,19]. The variability in the results of the Lovaas [14] study have been related to variability in the severity of the study participants’ ASD symptomatology, with participants with Pervasive Developmental Disorder, Not Otherwise Specified (PDD-NOS), a former diagnosis that included fewer symptoms than ASD, showing better outcomes than study participants that met full criteria for ASD [18]. 

Despite the positive impact of early intervention for preschoolers with ASD (age 12–72 mon), response to the intervention program is variable [18,20]. Outcomes for preschoolers who received early intervention range from loss of diagnosis to lack of improvement in the core ASD symptoms, from dramatic gains in language, cognitive, and adaptive skills to minimal treatment gains [21]. There are at least two possible reasons for the variability in the outcome of early-intervention studies. First, most studies do not describe the sample characteristics in detail. Even less is mentioned about the social and demographic factors that might influence the outcome [22]. Second, is the clinical heterogeneity of autism [23]. Despite the current custom of conceptualizing autism as a spectrum disorder following the publication of fifth edition of The Diagnostic and Statistical Manual of Mental Disorders (DSM-5) [1], it may be the case that subtypes exist within the autistic spectrum [24]. 

In addition to the possible subtypes of autism, several medical and behavioral conditions are known to co-exist with it. It is estimated that approximately 75% of individuals with ASD present with associated medical conditions, genetic syndromes, or mental health disorders [3,25]. On the other hand, in some studies, due to the attempt to recruit homogeneous samples of individuals with “pure” ASD, children with associated conditions such as epilepsy, severe intellectual disabilities, or genetic abnormalities, are not included [12]. Many studies also used small clinical samples or lacked details about the ASD characteristics that lead to diagnosis [22]. 

Thus, by excluding persons with ASD who have associated medical and behavioral disorders, who constitute the majority of the general ASD population [26], these stringent exclusion criteria significantly reduce the generalizability of results and reduce their utility in the real world. Without knowing the characteristics of the children who benefit from the intervention, it is difficult to make treatment recommendations in clinical practice. This review aimed to examine the exclusion criteria used in the early-intervention studies of ASD, in order to ascertain how these criteria are related to the efficacy of behavioral interventions for young children with ASD. 

## 2. Materials and Methods

Our review included 26 papers written between 2002 and 2018 that highlighted studies with three varying levels of exclusion criteria used in early behavioral interventions for children with ASD. PubMed and PsycINFO were the databases used to identify articles included in this review. Search terms used included various combinations of the following terms: “Early intervention”, “Autism”, “Autism Spectrum Disorder”, “children with autism”, “children with ASD”, “clinical trial”, and “group design”. A filter limiting the results to publication years of 2002 to 2018 was applied. Other studies were found from the reference list of the articles that met these inclusion criteria. The search was conducted through December 2018. 

The titles and abstracts of these studies were reviewed by the first, second, and fourth authors for appropriateness to include in the literature review, particularly for the inclusion of a behavioral intervention and the age of study participants. Inclusion criteria for this review were studies that (1) used participants between the ages of 2 and 5 years with Autism Spectrum Disorder, (2) investigated a behavioral intervention, (3) used a group design, and (4) were published within the last 15 years. Group studies were the focus of this review so that comparisons could be drawn among studies. Early behavioral intervention was another focus of this review, which is why studies that only used young participants and behavioral intervention were included. Given the increasing prevalence of ASD and corresponding treatments, the focus was also on studies recently published. Studies using both DSM-IV-TR [27] and DSM-5 [1] criteria were included, as there were few studies using DSM-5 criteria. Studies that employed single-case design and nonbehavioral interventions, such as dietary and pharmacological interventions, were excluded. No language filters were applied, but only one study was excluded for being in a language other than English. 

## 3. Results

There were 26 studies found based on the search methods and inclusion criteria specified above, published between the years of 2002 and 2018. For this review, the term “restrictive exclusion criteria” categorizes studies that excluded children with comorbidities and/or associated family mental health conditions. The term “loosely defined exclusion criteria” defined studies that included children with comorbidities but excluded certain individuals on the basis of other factors, such as distance of the family from the treatment center, non-English-speaking participants, or severe sensory or motor deficits. The term “exclusion criteria not defined” highlighted studies that did not significantly excluded any children. A summary of all studies can be found in Table A1. 

### 3.1. Restrictive Exclusion Criteria

Of the studies, 57% (*n* = 15/26) used comparably restrictive exclusion criteria to select their participants. Studies with this type of restrictive criteria mainly excluded participants with medical conditions other than ASD, such as genetic syndromes, epilepsy, and intellectual impairments. 

Perera, Jeewandara, Seneviratne, and Guruge [28] investigated an early-intervention program for children aged 18 to 40 mon in Sri Lanka. Study participants were children who had just received an initial diagnosis of Autism, were 18 to 40 mon in age, and had never received behavioral or developmental intervention previously. Participants were excluded if they had a diagnosis of PDD-NOS or Asperger’s Disorder, had severe cognitive impairments, experienced co-occurring sensory or motor disorders, genetic disorders, or if they had participated in developmental intervention prior to joining the study. Experimental group participants received home-based therapy in which their mothers were taught to use developmental and behavioral interventions to use with their children. Participants in the comparison group had received a diagnosis of autism over the age of 40 mon and did not receive any autism-specific developmental intervention. This study did not use random assignment. Results indicated that the children in the experimental group showed more improvement on measures of autism severity and social interaction, despite some improvement in the children in the comparison group. 

Brian, Smith, Zwaigenbaum, and Bryson [29] conducted a cross-site, randomized, controlled trial investigating the efficacy of a parent-mediated intervention, social ABCs, for toddlers aged 16 to 30 mon with suspected or confirmed ASD. Exact numbers of male and female participants were not given. Inclusion criteria included children who met criteria for ASD or displayed behaviors consistent with ASD, did not spend more than half their time in childcare, were products of full-term delivery, and had a birthweight above 2500 g. Exclusion criteria included the occurrence of any co-occurring genetic, neurological, or severe sensory or motor conditions. Results indicated that children in the treatment group showed more gains in functional vocal responsiveness to parent prompts and child vocal initiation as compared to the control group. 

Rogers et al. [30] conducted a randomized controlled trial with 98 children (76 boys) aged 12 to 24 mon. The study strove to investigate the efficacy of the Early Start Denver Model (ESDM), which fosters parental involvement within a child-centered interactive context and may be compared to conventional community therapies. Inclusion criteria specified that the children met risk criteria for ASD in a clinical assessment, were ambulatory, had a development quotient of 35 or higher, and primarily spoke English at home. The exclusion criteria included children who had parents that self-reported mental illness or substance abuse, children who had significant medical conditions such as cerebral palsy, a gestational age of less than 35 weeks, and/or genetic disorders related to developmental disabilities, or individuals who had current or prior enrollment in an intensive 1:1 autism intervention curriculum for more than 10 h per week. The main outcomes of this study were that individuals who had received parental training with the ESDM technique established more productive working alliances with their therapists as compared to the community group. However, the effects seen in intensive-treatment studies were not observed. They demonstrated that younger age and greater intervention positively affected the developmental rates for children with autism.

Carter et al. [31] conducted a study with 62 children (51 boys) aged 15 to 25 mon. The study aimed to investigate the efficacy of Hanen’s More Than Words (HMTW), a parent-implemented intervention, as compared to a control group. The inclusion criteria required the children to meet the diagnostic criteria of ASD and to be recruited from ASD specialty clinics. Children with a genetic disorder, those who did not obtain a predetermined “at-risk” score on the Screening Tool for Children with Autism (STAT), or those who did meet the symptom criteria for an ASD diagnosis based on clinical evaluations were excluded. The main outcomes of this study were that the HMTW group showed differential effects on child communication. However, parents of children who possessed higher object interest may require additional support to implement proper strategies. 

Dawson et al. [12] evaluated the efficacy of the ESDM with a sample size of 48 children aged 18 to 30 mon. Exact numbers of male and female participants were not given, but the ratio of males to females was 3.5 to 1. The inclusion criteria for this randomized controlled trial stipulated that the children meet criteria for ASD on the Toddler Autism Diagnostic Interview and Autism Diagnostic Observation Schedule (ADOS), receive a clinical diagnosis for ASD based on DSM-IV criteria, reside within half an hour of the testing location, and demonstrate a willingness to participate in a two-year or greater intervention program. Children who had a neurodevelopmental disorder of known etiology, significant sensory or motor impairments, major physical problems such as chronic or serious health conditions, seizures at the time of entry, use of psychoactive medication, a history of serious head injury or neurological disease, alcohol or drug exposure during the prenatal period, or developmental quotient below 35 were excluded. The main outcomes of this study were that the children who received ESDM training demonstrated significant improvements in IQ scores and adaptive behavior and were more likely to have a change in diagnosis to pervasive developmental disorder. Moreover, the comparison group manifested greater delays in adaptive behaviors and demonstrated minimal improvement in baseline scores.

Kasari, Gulsrud, Wong, Kwon, and Locke [32] aimed to identify if a joint attention intervention would result in greater engagement between caregivers and toddlers with autism. The randomized controlled trial investigated 38 children (29 boys), aged 21 to 36 mon. Inclusion criteria stated that children must have met criteria for autism following DSM-IV criteria by an independent clinician; children with additional syndromes were excluded. The main outcomes were that both caregivers and toddlers in the experimental group made significant improvements in areas of joint engagement, including responsiveness to joint attention and diversity of functional play acts, as compared to the control group.

Zachor and Itzchak [33] compared the efficacy of applied behavior analysis (ABA) and the integration of several intervention approaches for children with varying levels of autism severity. The quasi-experiment investigated a sample size of 78 (71 boys), aged 15 to 35 mon. Participating children had to meet a clinical diagnosis of autism based on DSM-IV criteria and the cut-off points on the ADI-R (Autism Diagnostic Interview-Revised); those with additional major medical diagnoses or incomplete post-intervention assessments were excluded. While there were no significant between-group differences in terms of improved cognitive abilities or adaptive skills, Zachor and Itzchak demonstrated that in the group with less severe baseline ASD symptoms, the children who had received the eclectic intervention approach had better outcomes in communication and socialization adaptive skills.

Itzchak and Zachor [34] also sought to characterize the stability and changes of autism diagnosis in correlation with pretreatment predictors and post-intervention outcomes. The open-design study investigated a sample size of 68 (62 boys), aged 18 to 35 mon. Inclusion criteria required that the child met established DSM-IV criteria for autism. Exclusion criteria were comorbidities, including genetic syndromes and seizure disorders. The main outcomes of this experiment suggest that individuals who had a changed diagnostic classification to ASD or Off Spectrum had better receptive language scores, as well as significant improvements in cognitive outcomes, adaptive outcomes, and reduction of stereotyped behaviors, as compared to individuals within the unchanged classification group.

Kasari, Paparella, Freeman, and Jahromi [35] investigated the effects of joint attention (JA) and symbolic play (SP) behavioral interventions in accordance with prediction to language outcomes. The study analyzed a sample size of 46 boys, aged 36 to 48 mon. Inclusion criteria required that the children had been diagnosed with autism on the ADI-R and ADOS scale, had to be of 5 years of age or younger, and had to be accessible for follow-ups. Exclusion criteria included seizure disorder and additional medical diagnoses, such as genetic syndromes. The main outcomes of this experiment included greater JA and SP skills and ability to execute these skills during play, within the respective groups as compared to the control group.

Ben-Itzchak and Zachor [36] sought to understand the correlation between cognitive, socialization, and communication pre-intervention variables to outcome in children with autism post-intervention. The study investigated a sample size of 25 (23 boys), aged 20 to 32 mon. Inclusion criteria included children diagnosed using the ADI-R and ADOS protocols. Exclusion criteria included children who demonstrated comorbidities, including genetic syndromes and seizure disorders. The main outcomes of this experiment were that the children demonstrated significant improvements in imitation, receptive and expressive language, nonverbal communication, play skills, and stereotyped behaviors. 

Remington et al. [37] investigated the effects of early intensive behavioral intervention for children with autism. The quasi-experiment analyzed a sample size of 44, aged 30 to 42 mon. Exact numbers of male and female participants were not given. Inclusion criteria included that the children had to be diagnosed with autism based on the ADI-R, had a previous diagnosis of autism by a clinician independent of the research program, or had a suspected diagnosis of autism, to be between 30 and 42 mon of age at the time of induction, and had to live in their family home. The exclusion criteria included that the child had to be free of any other chronic or serious medical conditions that might interfere with the ability to deliver consistent intervention or might adversely affect development. The main outcomes included significant improvements in IQ scores, daily living skills, motor skills, and language abilities subsequent to the interventional therapies. Moreover, children who participated in the early behavioral intervention therapy were more likely to attend mainstream schools, as compared to children within the control group.

Zachor, Ben-Itzchak, Rabinovich, and Lahat [38] compared the Eclectic-Development (ED) and ABA intervention approaches in children with autism. The quasi-experiment analyzed a sample size of 39 (37 boys), aged 22 to 34 mon. Inclusion criteria included that the children were diagnosed with autism using the ADI, met established criteria for Autism/PDD-NOS according to DSM-IV criteria. Exclusion criteria included children who had medical abnormalities such as seizures or hearing deficiencies. The main outcomes of this experiment demonstrated that ABA intervention approaches provided children with greater improvements in language communication and social interaction, as well as allowed for greater changes in diagnostic classifications, as compared to ED intervention approaches. 

Cohen, Amerine-Dickens, and Smith [39] sought to investigate the effects of early intensive behavioral treatment (EIBT) for children with autism. The quasi-experiment utilized a sample of 42 (35 boys), aged 20 to 41 mon. Inclusion criteria included that children had a primary, previous, and psychological diagnosis of autistic disorder or pervasive development disorder confirmed by ADI-R, pretreatment IQ above 35 on the Bayley Scales of Infant Development-Revised (BSID-R), chronological age between 18 and 42 mon at diagnosis and under 48 mon at treatment onset, residence within 60 kilometers of the treatment agency, and parental agreement to active participation. Exclusion criteria included children who had a severe medical limitation or illness, including motor or sensory deficits, that would prevent a child from participating in treatment for 30 h a week, and children who had undergone more than 400 h of prior behavioral intervention. The main outcomes of this experiment suggested a significant difference in the IQ scores and adaptive behavior for children who had undergone the EIBT, and a significant increase in EIBT children in regular education as compared to the control group. However, there were no significant between-group differences in language comprehension or nonverbal skills.

Kasari, Freeman, and Paparella [40] examined the efficacy of JA- and SP-targeted interventions. The randomized controlled study investigated a sample size of 58 (46 boys), aged 36 to 48 mon. Inclusion criteria included that children had a diagnosis of autism on the ADI-R and ADOS, were of 5 years of age or younger, and were accessible for follow-ups. Exclusion criteria included no seizure disorders or additional medical diagnoses, and children whose parents demonstrated refusal of final assessments or who left the program unexpectedly. The main outcomes of this experiment demonstrated improvements of JA and SP within the respective experimental groups, as well as significantly greater growth in expressive language for the individuals within these groups.

Eikeseth, Hayward, Gale, Gitlesen, and Eldevik [41] investigated the outcomes of varying intensities of early behavioral intervention for children with autism. The open-design study initially analyzed a sample size of 23 (17 boys), aged 28 to 42 mon. Inclusion criteria included diagnosis of autism according to the ICD-10 (International Classification of Diseases), chronological age at intake between 24 and 42 mon, the absence of other severe medical conditions as certified by a medical practitioner, and if the child resided outside of the catchment area for the clinical-based services. Exclusion criteria included an increased intensity of supervision due to lack of acquisition (as was the case for one child). The main outcomes of this experiment demonstrated a correlation between the intensity of supervision with changes in IQ scores and visual-spatial IQ after 14 mon. However, there was no significant correlation with the intensity of supervision and adaptive functioning.

Many of the studies that fell within the restrictive exclusion criteria category demonstrated positive outcomes of early behavioral interventions on various developmental skills including autism severity, verbal communication, social interaction, and other markers of development in comparison to control groups. Thus, these studies demonstrated promising results in improvement of many skills for young children with ASD. However, the restrictive nature of these studies limits the applicability of their outcomes to a wider audience of children with ASD who present with some form of comorbidity. 

### 3.2. Loosely Defined Exclusion Criteria

Of the studies discussed in this review, 15% (*n* = 4/26) utilized loosely defined exclusion criteria for their early-intervention behavioral treatments. Studies with loosely defined criteria included children who experienced ASD with comorbidities but excluded subjects based on other factors, such as primary language and accessibility to testing sites, or severe motor or sensory deficits. 

Yoder and Stone [42] evaluated two different communication interventions: Responsive Education and Prelinguistic Milieu Teaching (RPMT) and the Picture Exchange Communication System (PECS) in preschool children with ASD. The randomized group experiment included 36 children with a diagnosis of ASD or PDD-NOS aged 18 to 60 mon, who demonstrated communication deficits and passed hearing screenings. Of the participating children, 31 were boys. Participants were excluded from the study if they demonstrated severe sensory or motor deficits or if English was not the primary language spoken in the home. Of the 120 children who were screened for participation in the study, only 60 met inclusion criteria. Results demonstrated mixed results, with RPMT demonstrating better effects with generalized turn taking and generalized joint attention initiation as compared to PECS. Conversely, PECS demonstrated better effects with generalized requests in children who arrived to the study with little initiation of joint attention. 

Oosterling et al. [43] strove to understand the efficacy of non-intensive parental training in combination with standard care for children with autism. The randomized, controlled trial investigated a sample size of 75 (52 boys), aged 12 to 24 mon. Inclusion criteria included children with a clinical diagnosis of ASD or PDD-NOS, a demonstrated developmental potential at 12 mon, and a developmental quotient below 80. Exclusion criteria included family problems that may interfere with parental training and insufficient parental proficiency in the native language, Dutch. The main outcomes of this experiment suggested that additional non-intensive parental training did not have any influence on language and global clinical improvement outcome variables.

Wetherby et al. [44] sought to compare the effects of two parent-implemented Early Social Interaction (ESI) interventions. The randomized, controlled trial investigated a sample size of 82, aged 16 to 20 mon. Exact numbers of male and female participants were not given, but the individual ESI group contained 81% male participants, and the group ESI contained 92.5% male participants. Inclusion criteria included children who had received an ASD diagnosis between ages 16 to 20 mon and lived within 50 miles of either research site. Exclusion criteria included children who demonstrated participation in other interventional research studies. The main outcomes demonstrated that children within the individual social intervention groups improved their social communication, daily living, receptive language, and social skills, while children within the group intervention groups demonstrated worsening or no significant change in these measures.

Howard, Sparkman, Cohen, Green, and Stanislaw [45] compared the effects of intensive behavior analytic intervention (IBT), intensive eclectic intervention, and non-intensive public early-intervention programs in children with autism. The quasi-experiment investigated a sample size of 61 (54 boys), all less than 48 mon of age. Inclusion criteria included children who were independently diagnosed with Autistic Disorder or PDD-NOS according to DSM-IV criteria, entry into an intervention program before 48 mon of age, English spoken as the primary language within the child’s home, no significant and separate medical condition, and no prior treatment of more than 100 h. Exclusion criteria included individuals who had not completed the 7 mon of intervention, and parents who could not be contacted to arrange follow-up testing despite repeated attempts or refusal of testing. The main outcomes of this trial demonstrated that individuals who participated in the IBT group performed significantly higher in tests for IQ, nonverbal and verbal language, overall communication, and social skills.

The studies that utilized loosely defined exclusion criteria provide a stronger foundation to apply certain early-intervention behavioral methods to a wider range of children with ASD, given that they included a more diverse participant pool. However, not only are there a limited number of studies available with this type of exclusion criteria, but the criteria were often so specific to the particular study that it inhibited any potential conclusions that may be drawn in understanding the applicability of these outcomes to a wider range of children with ASD. This could compromise the generalizability of the results of these studies to a wide range of children with ASD.

### 3.3. Exclusion Criteria Not Defined 

Of the studies discussed in this review, 30% (*n* = 7/26) did not specifically list any exclusion criteria for the participants of their early-intervention behavioral treatments, and thus, the results of these studies may be applied to the comparably widest range of children with ASD. 

Welterlin, Turner-Brown, Harris, Mezibov, and Delmolino [46] implemented the Treatment and Education of Autistic and Communication Related handicapped Children (TEACCH) program in home-based models for parents of toddlers with ASD. Inclusion criteria for the study were chronological age of less than 42 mon and a diagnosis of Autism. No other exclusion criteria were specified. Twenty children participated in the study and were randomly assigned to receive TEACCH intervention at home or wait-list control. Six children participated in the experimental group and, of these, five were male. Participants were matched for data analysis between the experimental and control groups on the basis of similar age. Results between the experimental and control group did not reach statistical significance, which the authors attributed to low sample size and short time frame. 

Reed, Osborne, and Corness [47] conducted a study of 33 children who were nonrandomly assigned to treatment groups. Inclusion criteria were as follows: Age of 2 years, 6 mon to 4 years, 0 mon at the start of their intervention, and a diagnosis of ASD. No details were given about the number of males and females that participated. The only exclusion criterion specified was that the children participating in the study must not have been involved in any other major intervention at the same time as the study. Children were divided into one of three treatment groups. One group received preschool special education, another received special education designed specifically for autism, and the final group received in-home one-on-one behavioral treatment. After 10 mon of intervention, results from the three groups were compared, with some improvement in measures used across both special education groups. Children in the home-based program showed improvement across the Psych-Educational Profile and British Abilities Scale, but not for the Vineland Adaptive Behavior Scales.

Smith, Flanagan, Garon, and Bryson [48] examined Pivotal Response Training (PRT) in an Early Intensive Behavioral Intervention (EIBI) program delivered in the community. Inclusion criteria for the study were: Having a diagnosis of Autism Spectrum Disorder and age below 6 years. Children who met eligibility criteria were randomly assigned to participate in the experimental group. No control group was used. Rather, participants were divided into subgroups for data analysis, based on their scores on measures of intellectual functioning. Results demonstrated that all study participants, regardless of cognitive functioning level, showed significant improvement in communication skills and adaptive functioning, with larger gains found for the children in the moderate and high cognitive functioning groups. 

Fernell et al. [49] conducted a naturalistic, prospective study with 208 children aged 1½ to 4½ years. No information was given on the number of males and females included in the study. Children included in the study had a previous diagnosis of Autism that was confirmed through further testing for inclusion in the study, but no exclusion criteria were given, beyond parents’ language proficiency in Swedish or English. All children in the study received some form of applied behavior analysis (ABA), and participants self-selected into intensive ABA or non-intensive ABA. There was no control group. This study showed that study participants improved in several areas of functioning, and participants in intensive intervention did not show more improvement than participants in non-intensive intervention.

Landa, Holman, O’Neill, and Stuart [50] evaluated the effects of a curriculum aimed to improve socially synchronous behaviors for children with autism. The randomized, controlled trial investigated a sample size of 48 (40 boys), aged 21 to 23 mon. The inclusion criteria specified that the children met criteria on the ADOS, received a diagnosis of ASD from an expert clinician, had a nonverbal mental age of at least 8 mon, had no siblings with ASD, English was the primary language spoken within the home, and no known etiology for ASD. No exclusion criteria were specifically listed. The main outcomes for this experiment included significant between-group differences for socially engaged imitation, but no significant between-group differences for shared positive affect, expressive language, or nonverbal cognition.

Ingersoll [51] evaluated the efficacy of Reciprocal Imitation Training (RIT) in development elicited and spontaneous imitation skills in children with autism. The randomized, controlled trial investigated a sample size of 21 (18 boys), aged 27 to 47 mon. The inclusion criteria mandated that the children receive a clinical diagnosis of autism based on DSM-IV-TR criteria and met the cut-off for ASD on ADOS. There were no exclusion criteria that were explicitly listed. The main outcomes for the experiment included significantly more gains in elicited and spontaneous imitation for both objects and gestures, as compared to the control.

Reed, Osborne, and Corness [52] investigated the efficacy of home-based early behavioral interventions for children with autism. The quasi-experiment investigated a sample size of 27 (27 boys), aged 31 to 48 mon. Children included in the study were within 2 years, 6 mon and 4 years of age, received no other major intervention during the period of assessment, and had a diagnosis of ASD. The exclusion criteria were not listed. The main outcomes of this experiment demonstrated significant between-group differences in educational functioning, with no significant between-group differences for intellectual functioning, adaptive behavior, and ASD severity. 

The studies described above that did not specifically exclude children from participating in the study present results that are generalizable to the broadest population of children with ASD. However, this same lack of any exclusionary criteria also prevents understanding the specific methods of treatment necessary for the many different types of children who are diagnosed with ASD. Thus, the wider generalizability leads to fewer conclusions that can be drawn about the applicability of these results to any one specific child.

## 4. Discussion

This review evaluated 26 early-intervention behavioral studies of ASD based on their exclusion criteria into three categories: Restrictive, loosely defined, and not defined. These categories carry critical implications, as these categories define which of their outcomes may be applied to various audiences of children with ASD.

There were 15 studies that utilized restrictive criteria risk excluding approximately 75% of children who have ASD with a comorbid condition, including the 10% with a co-occurring psychiatric disorder, and the 4% with a genetic or chromosomal disorder [53]. Others excluded children with common neurological conditions, such as fragile X syndrome or epilepsy, which are strongly associated with autism [25]. Prevalence of ASD in children with epilepsy is around 6.3% with higher prevalence up to 47% in children with other forms of seizure disorders [54]. Other studies excluded children born before 35 weeks, although some studies suggest that about 7% of preterm infants might develop autism [55]. These studies may exclude a large group of individuals with ASD. Although many of these studies categorized the children as having improved, the results suggest that interventions work only for the minority of children who have “pure” ASD. For this reason, it is not possible to conclude that early intervention works in all children with ASD. 

The four studies that utilized loosely defined exclusion criteria and the seven studies that did not define any exclusion criteria may have included children with comorbid disorders that could have influenced their findings. Indeed, these studies showed mixed results, with some experimental groups showing more improvement than control groups, and others showing no significant between-group differences. Inclusion of comorbidities makes these studies’ results more applicable to a wide range of children with ASD, but also makes it difficult to know which interventions might be efficacious for specific comorbidities with ASD, since inclusion of comorbidities was typically not limited to only specific disorders. 

We believe that studies that investigate behavioral interventions for young children with ASD should make more of an effort to recruit and include study participants with comorbid conditions in addition to ASD, which could make their results more applicable to a wider range of children with ASD. It will also be important for these comorbid conditions to be explicitly listed in the participant characteristics so that conclusions can be drawn about how efficacious certain behavioral interventions are for children with ASD and associated conditions. Listing the participants with these descriptors may make it easier to understand what population of children with ASD may be most likely to benefit from the interventions studied. 

Current guidelines suggest not to exclude individuals with associated conditions if these are common. Given the number and incidence of comorbid disorders it may be hard to try to identify individuals who only meet criteria for ASD and no other disorders. Moreover, this may not be representative of the population of children with ASD. This review highlights the possible influence of treatment modifiers such as comorbidity in the outcome of behavioral interventions for young children with ASD. Overall, the results suggest that the heterogeneity observed in the response to early behavioral intervention in children with ASD may be related to various comorbid conditions. They underscore the need to systematically screen for the presence of comorbid symptoms and conditions at the time of recruitment of subjects, identify these in their studies, and modify intervention methods accordingly. How those interventions should be modified remains unclear as there is not yet enough research evidence to suggest what are evidence-based interventions for ASD with comorbid conditions.

A supplementary table, depicting the studies included in this review, grouped by intervention type, is available in Table A2. 

## 5. Limitations

There are some important limitations in this literature review. To begin, this review only included studies that used a group design. This is an important limitation about the results of this review, given that many studies investigating a behavioral intervention for young children with ASD use single-subject research design [56], which has been increasing over recent years [57]. However, group study designs for investigating behavioral interventions for individuals with ASD are an important part of identifying evidence-based practices for ASD [58] and allow for decisions to be made about the efficacy of a particular intervention [57]. In addition, the research databases used (PsychINFO, PubMed) are widely used and represent many research studies, but they are not inclusive of all research being conducted, so it is possible that some studies that could have met this review’s inclusion criteria were missed.

## Appendix A

**Table A1:** Summary of early-intervention studies reviewed.

**Restrictive Exclusion Criteria** studies that excluded children with co-morbidities and/or associated family mental health conditions				
**Authors**	**Sample**	**Inclusion Criteria**	**Exclusion Criteria**	**Main Outcomes**
Perera, Jeewandara, Seneviratne, & Guruge (2016) [28]	62 children (48 boys) aged 18–40 mon	Children aged 18 to 40 mon and who were diagnosed with autism for the first time at intake and had not received developmental interventions of any form previously.	Exclusions: (i) those diagnosed with other pervasive developmental disorders and Asperger disorder, (ii) those with severe cognitive impairment with autistic features, (iii) those diagnosed with autism having associated motor and sensory disorders and genetic disorders, (iv) those who had received other developmental interventions before intake and during the course of the study, and (v) those who dropped out before completion of the intervention period.	Children in the experimental group showed more improvement on measures of autism severity and social interaction, despite some improvement in the children in the comparison group
Brian, Smith, Zwaigenbaum& Bryson (2017) [29]	62 children aged 16–30 mon	Children with either confirmed ASD diagnosis or elevated scores on measures that assess ASD symptoms, no more than half-time childcare, between 36 and 42 weeks’ gestation, birthweight >2500 g, and absence of identifiable neurological, genetic, or severe sensory or motor conditions	Not specifically listed, but 11 children were evaluated and determined not to fit study inclusion criteria, and additional child met inclusion criteria but dropped out of the study early and those results were not included in the analysis	Children in the experimental group showed significant gains over the control group in the following areas assessed: child functional vocal responsiveness to parent prompts, child vocal initiations, parent smiling, fidelity of implementation, and parent-reported self-efficacy
Rogers et al. (2012) [30]	98 children (76 boys) aged 12–24 mon	Children met risk criteria for ASD in a clinical assessment, were ambulatory, had a development quotient of 35 or higher, and primarily spoke English within the home	Children who had parents that self-reported mental illness or substance abuse, children who had significant medical conditions such as cerebral palsy, a gestational age of less than 35 weeks, genetic disorders related to developmental disabilities, or individuals who had current or prior enrollment in an intensive 1:1 autism intervention curriculum for more than 10 h per week	Individuals who had received parental training in the Early Start Denver Model technique established more productive working alliances with their therapists as compared to the community group, however, the effects seen in intensive-treatment studies were not observed. Younger age and greater intervention h positively affected the developmental rates for children with autism
Carter et al. (2011) [31]	62 children (51 boys) aged 15–25 mon	Children that met the criteria of being diagnosed with ASD, and were recruited from ASD specialty clinics	Children that had a genetic disorder, children who did not obtain a pre-determined “at-risk” score on the Screening Tool for Children with Autism (STAT), or children who did meet the symptom criteria for an ASD diagnosis based on clinical evaluations	The intervention model, HMTW, showed differential effects on child communication based on a baseline factor, but parents of children who possessed higher object interest may require additional support to implement the proper strategies
Dawson et al. (2010) [12]	48 children (3.5M:1F) aged 18–30 mon	Children must meet criteria for ASD on the Toddler Autism Diagnostic Interview and ADOS, receive a clinical diagnosis for ASD based on DSM-IV criteria, had to reside within half an hour of the testing location, and demonstrate a willingness to participate in a 2-year or greater intervention	Children who had a neurodevelopmental disorder of known etiology, significant sensory or motor impairments, major physical problems such as chronic or serious health conditions, seizures at the time of entry, use of psychoactive medication, history of serious head injury or neurological disease, alcohol or drug exposure during the prenatal period, or developmental quotient below 35	ESDM group demonstrated significant improvements in IQ and adaptive behavior and were more likely to have a change in diagnosis to PDD-NOS. Comparison group manifested greater delays in adaptive behaviors and demonstrated minimal improvement in baseline scores
Kasari, Gulsrud, Wong, Kwon, & Locke (2010) [32]	38 children (29 boys) aged 21–36 mon	Children must have met criteria for autism following DSM-IV criteria by an independent clinician	Children with additional syndromes	Experimental group made significant improvements in joint engagement, responsiveness and diversity of functional play acts, as compared to the control group
Zachor & Itzchak (2010) [33]	78 children (71 boys) aged 15–35 mon	Participating children had to meet a clinical diagnosis of autism based on DSM-IV criteria and the cut-off points on the ADI-R	Additional major medical diagnoses or incomplete post-intervention assessments	No significant between-group differences in improved cognitive abilities or adaptive skills; Group with less severe baseline ASD and received eclectic intervention had better outcomes in communication, socialization, and adaptive skills
Itzchak & Zachor (2009) [34]	68 children (62 boys) aged 18–35 mon	Child met established DSM-IV criteria for autism	Comorbidities, including genetic syndromes and seizure disorders	Group with changed diagnostic classification had better receptive language scores, significant improvements in cognitive and adaptive outcomes, reduction of stereotyped behaviors
Kasari, Paparella, Freeman, & Jahromi (2008) [35]	46 boys aged 36–48 mon	Children had been diagnosed with autism on the ADI-R and ADOS scale, 5 years of age or younger, and be accessible for follow-ups	Seizure disorder and additional medical diagnoses, such as genetic syndromes	Greater JA and SP skills, and ability to execute these skills during play, as compared to the control group
Ben-Itzchak & Zachor (2007) [36]	25 children (23 boys) aged 20–32 mon	Children diagnosed using the ADI-R and ADOS protocols	Children who demonstrated comorbidities, including genetic syndromes and seizure disorders	Children demonstrated significant improvements in imitation, receptive and expressive language, nonverbal communication, play skills, and stereotyped behaviors
Remington et al. (2007) [37]	44 children aged 30–42 mon	Diagnosed with autism based on the ADI-R, or a previous diagnosis of autism by a clinician independent of the research program, or suspected diagnosis of autism, between 30 and 42 mon of age, and live in their family home	Free of any other chronic or serious medical conditions that might interfere with the ability to deliver consistent intervention or might adversely affect development	Significant improvements in IQ, daily living skills, motor skills, and language abilities. Early behavioral intervention group more likely to attend mainstream schools, compared to control group
Zachor, Ben-Itzchak, Rabinovich, & Lahat (2007) [38]	39 children (37 boys) aged 22–34 mon	Children were diagnosed with autism using the ADI, met established criteria for autism/PDD-NOS according to DSM-IV criteria	Children that had medical abnormalities such as seizures of hearing deficiencies	ABA intervention group had greater improvements in language and social interaction greater changes in diagnostic classifications, as compared to ED intervention approaches
Cohen, Amerine-Dickens, & Smith (2006) [39]	42 children (35 boys) aged 20–41 mon	Previous diagnosis of autistic disorder or PDD-NOS confirmed by ADI-R, IQ above 35 on the BSID-R, chronological age between 18-42 mon at diagnosis and under 48 mon at treatment onset, residence within 60 kilometers of the treatment agency	Children that had a severe medical limitation or illness, including motor or sensory deficits, that would prevent a child from participating in treatment for 30 h a week, and children that had underwent more than 400 h of prior behavioral intervention	EIBT group had significant difference in IQ and adaptive behavior and a significant increase in attendance in regular education compared to the control group. No significant between-group differences in language comprehension or nonverbal skills
Kasari, Freeman, & Paparella (2006) [40]	58 children (46 boys) aged 36–48 mon	Children had a diagnosis of autism on the ADI-R and ADOS, were of 5 years of age or younger, and were accessible for follow-ups	No seizure disorders or additional medical diagnoses, and children whose parents demonstrated refusal of final assessments or who left the program unexpectedly	Improvements of JA and SP within the respective experimental groups, as well as significantly greater growth in expressive language for the individuals within these groups
Eikeseth, Hayward, Gale, Gitlesen, & Eldevik (2009) [41]	23 children (17 boys) aged 28–42 mon	Diagnosis of autism according to the ICD-10, chronological age between 24 and 42 mon, the absence of other severe medical conditions, and if the child resided outside of the catchment area for the clinical-based services	Included an increased intensity of supervision due to lack of acquisition (as was the case for one child)	Demonstrated a correlation between the intensity of supervision with changes in IQ and visual-spatial IQ after 14 mon. However, there was no significant correlation with the intensity of supervision and adaptive functioning
Loosely Defined Exclusion Criteria studies that included children with comorbidities but excluded on the basis of other non-diagnostic factors				
Yoder & Stone (2006) [42]	36 children (31 boys) aged 18–60 mon	A diagnosis of autistic disorder or PDD-NOS; chronological age of 18 to 60 mon; fewer than 10 words in communication samples; and passed hearing screenings	Child were excluded who demonstrated severe sensory or motor deficits or if English was not the primary language spoken in the home	RMPT group showed higher frequency of generalized turn taking and generalized initiating joint attention. PECS facilitated generalized requests more than the RPMT in children with very little initiating joint attention prior to treatment.
Oosterling et al. (2010) [43]	75 children (52 boys) aged 12–24 mon	Clinical diagnosis of ASD or PDD-NOS, and a demonstrated developmental potential at 12 mon, and a developmental quotient below 80	Family problems that may interfere with parental training and insufficient parental proficiency in the native language, Dutch	Additional non-intensive parental training did not have any influence on language and global clinical improvement outcome variables
Wetherby et al. (2014) [44]	82 children aged 16–20 mon	Received ASD diagnosis between ages 16 to 20 mon and lived within 50 miles of either research site	Participation in other interventional research studies	Individual intervention improved social communication, daily living, receptive language, and social skills, while group intervention participants demonstrated worsening or no significant changes
Howard, Sparkman, Cohen, Green, & Stanislaw (2005) [45]	61 children (54 boys) less than 48 mon of age	Independently diagnosed with autistic disorder or PDD-NOS according to DSM-IV, entry into an intervention program before 48 mon of age, English spoken as the primary language within the child’s home, no significant and separate medical condition, and no prior treatment of more than 100 h	Individuals who had not completed the 7 mon of intervention, and parents who could not be contacted to arrange follow-up testing despite repeated attempts or refusal of testing	Individuals who participated in the IBT group performed significantly higher in tests for IQ, nonverbal and verbal language, overall communication, and social skills
Exclusion Criteria Not Defined studies that have not significantly excluded any children				
Welterlin, Turner-Brown, Harris, Mezibov, & Delmolino (2012) [46]	20 children, 2–3 years, 5 males in experimental group	Chronological age of less than 42 mon and a clinical diagnosis of autism	None	Mixed results; Treatment group showed improvements in independent work skills and parent ability to structure environment for learning; but no between groups differences could be supported for developmental gains or parent stress
Reed, Osborne, & Corness, 2010 [47]	33 children aged 2.5 to 4 years	Children included were aged 2:6 to 4:0 years at the start of their intervention; receiving no other major intervention during the period of the assessment; and had to have a diagnosis of ASD given by an independent pediatrician	None	Moderate improvements for children in all 3 groups.
Smith, Flanagan, Garon, & Bryson (2015) [48]	118 children aged 2–5 years (86% boys)	Children were selected randomly for the intervention program by their ASD diagnosis and age below 6 years	None	Significant gains in key language and cognitive outcomes for all groups. Baseline cognitive scores significantly predicted 1-year outcomes
Fernell et al. (2011) [49]	208 children aged 20–54 mon	Children had existing ASD diagnoses, no other inclusion criteria specified	None	Vineland composite scores increased over the 2-year period for by the subgroup with normal cognitive functioning. There was no significant difference between the intensive and non-intensive groups
Landa, Holman, O’Neill, & Stuart (2010) [50]	48 children (40 boys) aged 21–23 mon	Children met criteria on ADOS for ASD, Diagnosis of ASD from an expert clinician, had a non-verbal mental age of at least 8 mon, had no siblings with ASD, English the primary language spoken, and no known etiology for ASD	None	Significant between-group differences for socially engaged imitation, but no significant between-group differences for shared positive affect, expressive language, or nonverbal cognition
Ingersoll (2010) [51]	21 children (18 boys) aged 27–47 mon	Children receive a clinical diagnosis of autism based on DSM-IV-TR criteria and met the cut-off for ASD on ADOS	None	Significantly more gains in elicited and spontaneous imitation for both objects and gestures, as compared to the control
Reed, Osborne, & Corness (2007) [52]	27 children (27 boys) aged 31–48 mon	Children were within 2 years, 6 mon and 4 years of age, received no other major intervention during the period of assessment, and had a diagnosis of ASD	None	Significant between-group differences in educational functioning, with no significant between-group differences for intellectual functioning, adaptive behavior, and ASD severity

**Table A2:** Studies reviewed grouped by intervention type.

**Interventions based on Applied Behavior Analysis** studies that investigated interventions based on the principles of ABA, such as early intensive behavioral intervention, TEACCH				
**Authors**	**Sample**	**Exclusion Criteria Category**	**Intervention Investigated**	**Main Outcomes**
Zachor & Itzchak (2010) [33]	78 children (71 boys) aged 15–35 mon	Restrictive	Applied behavior analysis	No significant between-group differences in improved cognitive abilities or adaptive skills; Group with less severe baseline ASD and received eclectic intervention had better outcomes in communication, socialization, and adaptive skills
Itzchak & Zachor (2009) [34]	68 children (62 boys) aged 18–35 mon	Restrictive	Early intensive behavioral intervention (EIBI) vs. eclectic therapies	Group with changed diagnostic classification had better receptive language scores, significant improvements in cognitive and adaptive outcomes, reduction of stereotyped behaviors
Ben-Itzchak & Zachor (2007) [36]	25 children (23 boys) aged 20–32 mon	Restrictive	Early intensive behavioral intervention (EIBI)	Children demonstrated significant improvements in imitation, receptive and expressive language, nonverbal communication, play skills, and stereotyped behaviors
Remington et al. (2007) [37]	44 children aged 30–42 mon	Restrictive	Early intensive behavioral intervention (EIBI)	Significant improvements in IQ, daily living skills, motor skills, and language abilities. Early behavioral intervention group more likely to attend mainstream schools, compared to control group
Zachor, Ben-Itzchak, Rabinovich, & Lahat (2007) [38]	39 children (37 boys) aged 22–34 mon	Restrictive	ABA intervention vs. eclectic therapies	ABA intervention group had greater improvements in language and social interaction greater changes in diagnostic classifications, as compared to ED intervention approaches
Cohen, Amerine-Dickens, & Smith (2006) [39]	42 children (35 boys) aged 20–41 mon	Restrictive	Early intensive behavioral intervention (EIBI)	EIBT group had significant difference in IQ and adaptive behavior and a significant increase in attendance in regular education compared to the control group. No significant between-group differences in language comprehension or nonverbal skills
Eikeseth, Hayward, Gale, Gitlesen, & Eldevik (2009) [41]	23 children (17 boys) aged 28–42 mon	Restrictive	Early intensive behavioral intervention (EIBI)	Demonstrated a correlation between the intensity of supervision with changes in IQ and visual-spatial IQ after 14 mon. However, there was no significant correlation with the intensity of supervision and adaptive functioning
Howard, Sparkman, Cohen, Green, & Stanislaw (2005) [45]	61 children (54 boys) less than 48 mon of age	Loosely-defined	Early intensive behavioral intervention (EIBI) vs. eclectic therapies	Individuals who participated in the IBT group performed significantly higher in tests for IQ, nonverbal and verbal language, overall communication, and social skills
Welterlin, Turner-Brown, Harris, Mezibov, & Delmolino (2012) [46]	20 children, 2–3 years, 5 males in experimental group	Not defined	TEACCH	Mixed results; Treatment group showed improvements in independent work skills and parent ability to structure environment for learning; but no between groups differences could be supported for developmental gains or parent stress
Reed, Osborne, & Corness, 2010 [47]	33 children aged 2.5 to 4 years	Not defined	ABA therapy vs. normal educational practice	Moderate improvements for children in all 3 groups.
Smith, Flanagan, Garon, & Bryson (2015) [48]	118 children aged 2–5 years (86% boys)	Not defined	Pivotal Response Training	Significant gains in key language and cognitive outcomes for all groups. Baseline cognitive scores significantly predicted 1-year outcomes.
Fernell et al. (2011) [49]	208 children aged 20–54 mon	Not defined	Early intensive behavioral intervention (EIBI)	Vineland composite scores increased over the 2-year period for by the subgroup with normal cognitive functioning. There was no significant difference between the intensive and non-intensive groups
Reed, Osborne, & Corness (2007) [52]	27 children (27 boys) aged 31–48 mon	Not defined	Early intensive behavioral intervention (EIBI)	Significant between-group differences in educational functioning, with no significant between-group differences for intellectual functioning, adaptive behavior, and ASD severity
**Early Start Denver Model**				
Rogers et al. (2012) [30]	98 children (76 boys) aged 12–24 mon	Restrictive	ESDM	Individuals who had received parental training in the Early Start Denver Model technique established more productive working alliances with their therapists as compared to the community group, however, the effects seen in intensive-treatment studies were not observed. Younger age and greater intervention h positively affected the developmental rates for children with autism
Dawson et al. (2010) [12]	48 children (3.5M:1F) aged 18–30 mon	Restrictive	ESDM	ESDM group demonstrated significant improvements in IQ and adaptive behavior and were more likely to have a change in diagnosis to PDD-NOS. Comparison group manifested greater delays in adaptive behaviors and demonstrated minimal improvement in baseline scores
**Joint Attention and Symbolic Play Interventions**				
Kasari, Gulsrud, Wong, Kwon, & Locke (2010) [32]	38 children (29 boys) aged 21–36 mon	Restrictive	Joint attention intervention	Experimental group made significant improvements in joint engagement, responsiveness and diversity of functional play acts, as compared to the control group
Kasari, Paparella, Freeman, & Jahromi (2008) [35]	46 boys aged 36–48 mon	Restrictive	Joint attention and symbolic play	Greater JA and SP skills, and ability to execute these skills during play, as compared to the control group
Kasari, Freeman, & Paparella (2006) [40]	58 children (46 boys) aged 36–48 mon	Restrictive	Joint attention and symbolic play	Improvements of JA and SP within the respective experimental groups, as well as significantly greater growth in expressive language for the individuals within these groups
**Interventions Primarily Targeting Speech and Language**				
Carter et al. (2011) [31]	62 children (51 boys) aged 15–25 mon	Restrictive	Hanen’s More Than Words	The intervention model, HMTW, showed differential effects on child communication based on a baseline factor, but parents of children who possessed higher object interest may require additional support to implement the proper strategies
Yoder & Stone (2006) [42]	36 children (31 boys) aged 18–60 mon	Loosely-defined	Responsive Education and Prelinguistic Milieu Training (RPMT) and Picture Exchange Communication System (PECS)	RMPT group showed higher frequency of generalized turn taking and generalized initiating joint attention. PECS facilitated generalized requests more than the RPMT in children with very little initiating joint attention prior to treatment.
**Parent-Mediated Behavioral Interventions**				
Perera, Jeewandara, Seneviratne, & Guruge (2016) [28]	62 children (48 boys) aged 18–40 mon	Restrictive	Home-based intervention implemented primarily by participants’ mothers	Children in the experimental group showed more improvement on measures of autism severity and social interaction, despite some improvement in the children in the comparison group
Brian, Smith, Zwaigenbaum& Bryson (2017) [29]	62 children aged 16–30 mon	Restrictive	Social ABC’s, parent intervention	Children in the experimental group showed significant gains over the control group in the following areas assessed: child functional vocal responsiveness to parent prompts, child vocal initiations, parent smiling, fidelity of implementation, and parent-reported self-efficacy
Oosterling et al. (2010) [43]	75 children (52 boys) aged 12–24 mon	Loosely-defined	Parent intervention targeting joint attention	Additional non-intensive parental training did not have any influence on language and global clinical improvement outcome variables
Wetherby et al. (2014) [44]	82 children aged 16–20 mon	Loosely-defined	Parent-implemented social intervention	Individual intervention improved social communication, daily living, receptive language, and social skills, while group intervention participants demonstrated worsening or no significant changes
**Uncategorized Behavioral Interventions** studies that do not fit with any of the other categories				
Landa, Holman, O’Neill, & Stuart (2010) [50]	48 children (40 boys) aged 21–23 mon	Not defined	Interpersonal Synchrony or Non-Interpersonal Synchrony	Significant between-group differences for socially engaged imitation, but no significant between-group differences for shared positive affect, expressive language, or nonverbal cognition
Ingersoll (2010) [51]	21 children (18 boys) aged 27–47 mon	Not defined	Reciprocal Imitation Training	Significantly more gains in elicited and spontaneous imitation for both objects and gestures, as compared to the control
